# Burnout, lifestyle and relaxation among dentists in Lithuania: a cross-sectional study

**DOI:** 10.1186/s12913-021-07074-z

**Published:** 2021-10-15

**Authors:** Eglė Slabšinskienė, Andrej Gorelik, Aistė Kavaliauskienė, Apolinaras Zaborskis

**Affiliations:** 1 Medical Academy, Faculty of Odontology, Department of Oral Health and Pediatric Dentistry, Lithuanian University of Health Sciences, Kaunas, Lithuania; 2 Academy of Medicine, Faculty of Odontology, Department of Oral Health and Pediatric Dentistry, Lithuanian University of Health Sciences, J.Luksos-Daumanto street, 6, LT-50106 Kaunas, Lithuania; 3 Medical Academy, Faculty of Odontology, Department of Orthodontics, Lithuanian University of Health Sciences, Kaunas, Lithuania; 4 Medical Academy, Faculty of Public Health, Department of Preventive Medicine, Lithuanian University of Health Sciences, Kaunas, Lithuania

**Keywords:** dentists, burnout, lifestyle, relaxation, substances, Lithuania

## Abstract

**Background:**

The aim of this study was to estimate the association of burnout level with lifestyle and relaxation among dentists in Lithuania. A better understanding of this association could help in the development of targeted interventions to prevent burnout among these professionals.

**Methods:**

The survey was conducted among practising dentists (*N* = 380) using the Maslach Burnout Inventory (MBI) and an authors’ proposed scale to measure lifestyle and relaxation. Poisson regression was applied to examine the association between variables.

**Results:**

Regular cigarette smoking among dentists in Lithuania was 16.8 % and alcohol consumption was 31.3 %. Some forms of active relaxation were also common: regular playing sports (57.9 %), and spending time in nature (61.4 %). Emotional exhaustion (EE) and depersonalization (DP) burnout dimensions were negatively related to the regular use of illegal substances, alcohol, medication and smoking, while personal achievement (PA) was negatively related to smoking only. Dentists who regularly exercised had significantly lower EE and DP sum scores, and better assessments of PA. There were also positive relationships of EE, DP and PA sum scores with the variables of relaxation (e.g., spending time with family or friends, visiting a theatre, engaging in art, listening to music).

**Conclusions:**

It was concluded that the burnout dimensions are negatively associated with unhealthy lifestyle factors and positively associated with active relaxation among dentists in Lithuania. Therefore, burnout prevention should target specific lifestyle and relaxation improvement strategies.

## Background

Professional burnout is a multifaceted psychological syndrome characterized by varying degrees of emotional exhaustion (EE), depersonalization (DP), and low sense of personal accomplishment (PA) [1]. The key emerging factors of this syndrome are chronic interpersonal stressors at the job [2]. A high degree of burnout places individuals at increased risk of potentially serious personal and professional consequences indicating that these individuals are not “healthy” [3, 4]. Subsequently, the costs and consequences of burnout have attracted increasing attention from researchers [5, 6], so burnout is an increasing concern in today’s workplaces [7].

Burnout among workers in the healthcare sector has been studied quite extensively, so various preventive strategies have been proposed to combat job burnout among these professionals [8]. Systematic reviews of interventions primarily highlighted the individual strategies that included lifestyle change, relaxation training and task restructuring at work [3, 4, 9–12]. It has also been argued that energy balance-related behavior, including physical activity and sedentary and dietary behavior, may play an important role in preventing and/or curing burnout [5]. Ross et al. (2017) in their research on professional burnout among nurses demonstrated that initiatives to increase weekly exercising and information provision on healthy lifestyles, nutrition, and adequate sleep have direct relevance to the exhaustion component of burnout [13]. Verhavert et al. (2020) in their recent systematic literature review found nine out of ten experimental studies that showed the effectiveness of exercise programs in reducing burnout risk [5]. According to the review of the same authors, 14 out of 15 observational studies showed a negative association between physical activity and burnout risk. A few observational studies linking sedentary and dietary behavior with burnout risk suggested that being more sedentary and eating less healthy were each associated with higher burnout risk [14, 15]. The burnout of health professionals often coexists with negative emotions that lead to negative lifestyle choices [16, 17]. There are several studies that have examined the link of burnout with alcohol and tobacco use but their results were contradictory [18–21].

According to the studies presented, lifestyle and relaxation choices seem to play an important role in burnout. However, the findings in this research field are inconsistent, possibly because the major health surveys have been completed in different professional groups and there is little consensus regarding indicators for lifestyle and relaxation measurement. Moreover, there is very little research that has evaluated the association of burnout with lifestyle and relaxation among dentists.

In Lithuania, after the restoration of the country’s independence in 1991, dentistry very rapidly transitioned from a free-of-charge to a free market (paid) dental care delivery system that became predominantly driven by the private sector [22]. Currently, most dentists work in private dental clinics or own a small, at most one chair, private dental practice [23]. Commercial issues started to play a significant role in dentistry, while at the same time there were insistent constraints and control by government, sanitary and other institutions. Against this backdrop, practising dentists in Lithuania started to receive multiple stresses while carrying out clinical, administrative and managerial tasks [22]. Moreover, dentistry remains a very stressful profession with ever-changing technologies and methods of practice, with high expectations from oneself as well as patients. Multiple stresses lead to a high risk of burnout [24, 25]. Studies have confirmed that dentists were significantly more likely to experience burnout symptoms than any other medical profession group [25, 26]. A recently published article showed that the prevalence of burnout among Lithuanian dentists was also high [27].

The present study aimed to estimate the association of burnout level with different lifestyle and relaxation variables among dentists in Lithuania. A better understanding of this association could help in the development of targeted interventions to combat job burnout among these professionals.

## Methods

### Study design, participants, and data collection

 The present study is part of an observational population-based research project on burnout among Lithuanian dentists, which followed a cross-sectional design. The project conformed to the principles outlined in the World Medical Association Declaration of Helsinki. Ethical approval of the research project was granted by the Bioethics Committee Center of the Lithuanian University of Health Sciences on 16 October, 2019 (Protocol number: BEC–OF–13). The license to reproduce the required number of MBI copies was purchased from the copyright holder (Mind Garden, Inc., Menlo Park, CA, USA). The methods, including sampling, participants and data collection, have been described in detail in previous publications [27, 28]. Therefore, only specific methods for this study are presented.

The software G*Power 3.1 (University of Dusseldorf, Dusseldorf, Germany) [29] calculated a minimum sample of 260 participants, based on a z test in the Poisson regression model [30, 31] to assess the relationship between the MBI score and binomial predictors (π = 0.5), an alpha error of 5 %, a power of 80 %, a mean MBI score of 10, and an effect size to be detected of at least 1.1. Assuming that only approximately 50 % of the dentists in the initial sample would participate in the study, the sample size was increased to 520.

Data collection was carried out in two ways. In the first way, the survey was conducted among dentists who attended scientific conferences organized by the Lithuanian Dental Chamber in 5 regional divisions in October – December 2019. The attendance of these conferences is regulated by the licensing conditions for dentists and supported by the Lithuanian Dental Chamber; therefore, participation in these events is active among dentists in all regions of Lithuania. With the consent of the conference organizers, 400 dentists were randomly selected (80 attendees at each conference), and at the registration desk, they were invited to fill in the hard copy of the questionnaire. Of them, 246 (61.5 %) dentists agreed to participate in the study and completed the questionnaire. As the number of subjects was still insufficient, data collection was continued in a second way by conducting an online survey in January – February 2020. The questionnaire was shared in closed facebook groups “Odontologijos profesionalai“ (“Professionals of Odontology“) and ”Lietuvos odontologai” (“Dentists of Lithuania”) with limited access destined only to its members. Those who completed the questionnaire during the conferences were informed not to complete it. In this way, another 150 completed questionnaires (all purchased online copies) were received; however, the response rate for this group of respondents remained unknown because the number of Facebook account users was unreported.

Altogether, in both subsamples, 396 dentists participated in the study. Comparison of these groups according to demographic and the main research characteristics (e.g., burnout level) did not show statistically significant differences, so both groups of respondents were combined into one group, and the data were analyzed together. Since 16 respondents did not answer all MBI questions they were excluded; therefore, the final study sample comprised of 380 dentists.

### Measures

To evaluate levels of burnout among dentists, the MBI-HSS (Maslach Burnout Inventory – Human Services Survey, hereinafter referred to as MBI) was chosen as it is considered to be the most trustworthy and the most commonly used tool of this kind [32]. The instrument was translated into Lithuanian and was validated previously in other fields of medicine [33, 34]. In our recent study, we conducted factorial validation of the MBI among Lithuanian dentists, which showed that the MBI offers factorial validity and has an invariant structure across demographic and workload groups [28].

The inventory comprised 22 items with responses rated on a 7-point Likert scale ranging from 0 (“never”) to 6 (“daily”). The MBI included three subscales (dimensions): emotional exhaustion (EE; 9 items with sum score range of 0–54) measuring the feelings of being emotionally overextended and exhausted by one’s work; depersonalization (DP; 5 items with sum score range of 0–30) measuring the unfeeling and impersonal response toward recipients of one’s service, care treatment, or instruction; and personal accomplishment (PA; 8 items with sum score range of 0–48) measuring the feelings of competence and successful achievement in one’s work [32]. In analyses, the items of PA were reverse-scored, hence higher item scores implied higher level of burnout (higher emotional exhaustion and depersonalization, and lower personal accomplishment).

Information about the participants was also collected and included age, gender, professional and other characteristics. As this study focused on lifestyle and relaxation, the relevant information was collected with an authors’ proposed scale (see Results). The scale consisted of 15 items. Response categories were: “daily” (1), “several times a week” (2), “several times a month” (3), “several times a year” (4), and “never” (5). In analyses, the items were dichotomized by merging the categories “daily” (1) and “several times a week” (2) into a “regular” category and merging the remaining categories into an “irregular” category (e.g., smoking cigarettes regularly or irregularly). Based on the results of exploratory factor analysis, the items were divided into three groups: (1) use of substances (4 items); (2) passive rest (6 items); and (3) relaxation hobbies (5 items).

### Statistical analysis

Three subscale sum scores of the MBI (EE, DP, and PA) were calculated and considered continuous dependent variables in the Poisson regression. Meanwhile, the items of lifestyle and relaxation scale were considered independent variables (predictors). These variables were applied in binary form using the “regular” category as the contrast category and the “irregular” category as the reference category. The strength of the relationship between the sum score and the predictor was assessed by the ratio of sum score means (RSSM), which shows how many times the mean of the outcome variable (sum score) increases as the predictor changes from the reference category to the contrast category [30, 31].

The model fit to the existing data was assessed by deviance/degree of freedom; values close to 1 indicated a good model fit [30, 31]. The model was fitted and ran separately for each domain (EE, DP, and PA) using three sets (use of substances, passive rest, and relaxation hobbies as they were identified from exploratory factor analysis) of predictors, which resulted in 9 regression models. Such an approach allowed the identification of the factors most relevant to the burnout dimensions in each set of predictors. This facilitated the interpretation of the results. The models were tested adjusting the data for the gender and age of the study participants.

All analyses were performed using the SPSS statistical package (version 21; IBM SPSS Inc., Chicago, IL, 2012). Reported *P* values ≤ 0.05 indicated a statistically significant relationship (in tables these values are highlighted). We followed the Strengthening the Reporting of Observational Studies in Epidemiology (STROBE) cross sectional checklist when writing this article [35].

## Results

The study population comprised 380 dentists, 58 (15.3) males and 322 (84.7) females; 229 (60.3 %) dentists were 30 or older, and 176 (46.3 %) had 10 or more years of professional experience. By specialty, 278 (73.7 %) study participants were general dentists and 102 (26.3 %) had acquired postgraduate qualification.

The lifestyle and relaxation measure prevalence (frequency and percentage of reports “daily” or “several times a week”) among study participants is presented in Table 1. The most commonly used strategy for relaxation of dentists was passive rest, which was characterized by spending time with family or friends (89.3 %), sleeping (84.7 %), spending time on social media (83.1 %), eating some favorite food (86.8 %), watching TV (73.9 %), and listening to music (80.0 %). Some forms of active relaxation were also common: playing sports (57.9 %), and spending time in nature (61.4 %). Regular cigarette smoking among dentists in Lithuania was 16.8 %, and alcohol consumption was 31.3 %. Gender differences were significant in smoking prevalence (males 41.7 %, females 12.4 %, *P* < .001) and in rate of reading nonmedical literature (males 36.2 %, females 52.0 %, *P* = .045), while age differences (comparing two age groups) were significant in rates of spending time on social media (up to 30 years 94.6 %, 30 and more years 75.3 %, *P* < .001) and reading non-medical literature (up to 30 years 42.3 %, 30 and more years 54.8 %, *P* = .028).

**Table 1:** The percentage of the respondents who rated “daily” or “several times a week” the items on lifestyle and relaxation (*N* = 380)

Item	*n*	(%)
*Use of substances*:		
I use drugs (illegal substances)	20	5.3
I smoke cigarettes	64	16.8
I drink alcohol	119	31.3
I take medications to reduce stress or improve sleep	51	13.4
*Passive rest*:		
I spend time with family or friends	339	89.3
I sleep in my free time	322	84.7
I spend time on social media (Internet, Facebook, etc.)	316	83.1
I eat some food I like	330	86.8
I watch TV	281	73.9
I listen to music	304	80.0
*Relaxation hobbies*:		
I go to cultural events (theatre, concerts, etc.)	63	16.6
I engage in art (drawing, playing musical instruments, attending choir, etc.)	87	22.9
I play sports	220	57.9
I spend time in nature (camping, hunting, fishing, walking, etc.)	233	61.4
I read nonmedical literature	189	49.7

Statistical characteristics and Cronbach’s alpha of the MBI dimensions as well as the whole inventory are presented in Table 2. The average score per item (mean/number of items) indicated that respondents were likely to report higher scores for items of the EE dimension than for items of the PD and PA dimensions. Kolmogorov-Smirnov’s test for normality of the distribution of sum score was not significant for the EE dimension but was significant for the PD and PA dimensions. This finding was important in choosing the Poisson regression method of analysis of burnout relationship with other factors. Assessments of Cronbach’s alpha ranged from 0.737 for the DP dimension to 0.904 for the EE dimension, indicating acceptable to good internal consistency reliability of the MBI dimensions (this assessment for the whole inventory was 0.896).

**Table 2:** Main characteristics of the Maslach Burnout Inventory (*N* = 380)

Dimension	Number of items	Sum score				Cronbach’s alpha
		Mean	Mean/number of items	Standard deviation	*P* Test for normality^a^	
Emotional exhaustion	9	24.27	2.70	11.66	0.163	0.904
Depersonalization	5	7.78	1.56	5.94	**< 0.001**	0.737
Personal accomplishment	8	12.44	1.56	7.66	**0.011**	0.789
All dimensions	22	44.49	2.02	20.22	0.264	0.896

Note: ^a^ Kolmogorov-Smirnov test

The relationships between EE, DP and PA sum scores and the three groups of lifestyle and relaxation variables (use of substances, passive rest, and relaxation hobbies) were analyzed by multiple Poisson regression models adjusting data for gender and age of respondents.

Dentists who reported regular (“every day” or “several times a week”) use of illegal substances, smoking, use of alcohol, taking medications to reduce stress or improve sleep had a significantly higher mean EE score by 1.14 to 1.40 times compared with those who did not use these substances (Table 3). There was a significant interaction between the regular use of illegal substances and taking medications to reduce stress or improve sleep. In light of this interaction, it was found that individuals who reported regular use of both illicit substances and medications had significantly less (almost two times lower) emotional exhaustion. Similar figures were also observed in the analysis of DP sum scores. Personal achievement sum score had a significant relationship only with regular smoking, which showed that such smokers were more likely to report lowered rate of personal accomplishments than nonsmokers (in present analysis, the items of PA dimension were reverse-scored, so the mean of PA sum score among regular smokers was higher than among nonsmokers).

**Table 3:** Relationship between emotional exhaustion, depersonalization and personal accomplishment sum scores, and the individual use of substances (*N* = 380)

Item	Emotional exhaustion			Depersonalization			Personal accomplishment		
	RSSM	(95 % CI)	*P*	RSSM	(95 % CI)	*P*	RSSM	(95 % CI)	*P*
I use drugs (illegal substances)	1.35	(1.06–1.71)	**< 0.001**	2.16	(1.52–3.05)	**< 0.001**	0.87	(0.57–1.33)	0.510
I smoke cigarettes	1.27	(1.18–1.35)	**< 0.001**	1.35	(1.21–1.51)	**< 0.001**	1.41	(1.28– 1.55)	**< 0.001**
I drink alcohol	1.14	(1.08–1.20)	**< 0.001**	1.21	(1.11–1.32)	**< 0.001**	0.99	(0.92–1.07)	0.858
I take medications to reduce stress or improve sleep	1.40	(1.31–1.50)	**< 0.001**	1.52	(1.35–1.71)	**< 0.001**	1.09	(0.97–1.21)	0.137
I use drugs (illegal substances) & I take medications to reduce stress or improve sleep	0.55	(0.42–0.72)	**< 0.001**	0.33	(0.22 − 0.49)	**< 0.001**	1.19	(0.75–1.88)	0.455
Deviance/degree of freedom	5.05			4.24			4.38		

Note: RSSM (95 % CI) – ratio of sum score means adjusted for age and gender (95 % confidence interval)

There was also some significant relationship of EE, DP and PA sum scores with the variables of individual passive rest (Table 4). Spending time with family or friends, eating of preferred food, and listening to music were related to the EE sum score, diminishing emotional exhaustion of dentists. In this analysis, all kinds of passive rest, except regular watching TV, also had a positive impact on the depersonalization of individuals. Some of these items also had a positive relationship with the personal accomplishment of dentists, while watching TV had a negative effect on this dimension.

**Table 4:** Relationship between emotional exhaustion, depersonalization and personal accomplishment sum scores, and the variables of individual passive rest (*N* = 380)

Item	Emotional exhaustion			Depersonalization			Personal accomplishment		
	RSSM	(95 % CI)	*P*	RSSM	(95 % CI)	*P*	RSSM	(95 % CI)	*P*
I spend time with family or friends	0.88	(0.79–0.98)	**0.016**	0.80	(0.67–0.95)	**0.011**	0.75	(0.65–0.86)	**< 0.001**
I sleep in my free time	0.94	(0.87–1.01)	0.090	0.87	(0.77–0.99)	**0.032**	1.08	(0.97– 1.20)	0.188
I spend time on social media (Internet, Facebook, etc.)	1.05	(0.97–1.14)	0.219	1.22	(1.06–1.41)	**0.007**	1.09	(0.97–1.22)	0.167
I eat food I like	0.91	(0.82–1.00)	**0.046**	0.73	(0.62–0.85)	**< 0.001**	0.72	(0.63–0.82)	**< 0.001**
I watch TV	0.99	(0.94–1.05)	0.991	1.09	(0.99–1.19)	0.088	1.10	(1.02–1.19)	**0.017**
I listen to music	0.91	(0.86–0.96)	**0.002**	0.80	(0.73–0.89)	**< 0.001**	0.97	(0.89–1.05)	0.429
Deviance/degree of freedom	5.66			4.30			4.31		

Note: RSSM (95 % CI) – ratio of sum score means adjusted for age and gender (95 % confidence interval)

In analyses of another group of relaxation symptoms (Table 5), it was found that dentists who regularly played sports had significantly lower EE and DP sum scores, as well as better assessments of personal accomplishment. Other relaxation hobbies, such as regular going to cultural events, spending time in nature and reading nonmedical literature, also had a positive effect in weakening the dimensions of burnout.

**Table 5:** Relationship between emotional exhaustion, depersonalization and personal accomplishment sum scores, and the variables of individual relaxation hobbies (*N* = 380)

Item	Emotional exhaustion			Depersonalization			Personal accomplishment		
	RSSM	(95 % CI)	*P*	RSSM	(95 % CI)	*P*	RSSM	(95 % CI)	*P*
I go to cultural events (theatre, concerts, etc.)	0.88	(0.82–0.94)	**< 0.001**	0.90	(0.80–1.02)	0.094	1.07	(0.98–1.18)	0.136
I engage in art (drawing, playing musical instruments, attending choir, etc.)	1.01	(0.95–1.07)	0.818	1.0 2	(0.92–1.13)	0.718	0.86	(0.79– 0.94)	**0.001**
I play sports	0.83	(0.79–0.87)	**< 0.001**	0.89	(0.82–0.96)	**0.004**	0.89	(0.83–0.95)	**< 0.001**
I spend time in nature (camping, hunting, fishing, walking, etc.)	0.98	(0.93–1.03)	0.398	0.92	(0.85–1.00)	**0.049**	0.90	(0.84–0.96)	**0.001**
I read nonmedical literature	0.98	(0.93–1.02)	0.291	0.94	(0.87–1.03)	0.176	0.86	(0.81–0.93)	**< 0.001**
Deviance/degree of freedom	5.49			4.57			4.44		

Note: RSSM (95 % CI) – ratio of sum score means adjusted for age and gender (95 % confidence interval)

## Discussion

This study is an extension of research on job-related burnout among dentists in Lithuania. In our recently published article, we showed that the prevalence of burnout among Lithuanian dentists is high and is related to demographic and job-related factors [27]. Consequently, the present study aimed to estimate the association of burnout level with different lifestyle and relaxation variables among dentists in Lithuania. The results confirmed significant associations of the MBI dimensions with several factors of lifestyle and relaxation in Lithuanian dentists.

The findings of the study were based on survey data that were conducted among practising dentists in Lithuania. The sample was representative of demographic and professional characteristics of the Lithuanian dentists’ trade union, so these facts indicated the validity of the empirical data. Moreover, internal consistency reliability for the whole MBI and its dimensions ranged from acceptable to good and were in line with corresponding estimations in other similar studies [36–38]. The response rate in studies of burnout among dental practitioners ranged from 87.5 % [26] to 48 % [36]. In our study, 61.5 % of primary selected dentists agreed to participate in the study and completed the questionnaire while attending scientific conferences, so the response rate was comparable.

From a methodological point of view, it is important to choose an appropriate method for assessing the relationship between variables [30]. In our study, Poisson regression was chosen for this purpose. This choice was due to several reasons. First, not all MBI dimensions had a sum score that satisfied the normality of distribution. Second, the relationship between variables could be more precisely assessed if the model had to be multivariate. Third, and most importantly, the literature states that burnout is best considered a continuum rather than a dichotomous variable; as of now, the diagnostic criteria have not been well specified, and the necessary clinical research has not been done [1, 32, 39]. Indeed, the best analytic approach is to evaluate the relationship between symptoms of burnout and outcomes using individual domain sum scores as continuous data. The Poisson regression method satisfies all these requirements. In addition, we were able to use independent variables in dichotomized form, which facilitated the interpretation of the results.

The lifestyle characteristics of surveyed dentists were not much different from those of the university-educated general Lithuanian population by the prevalence of regular use of alcohol (31.3 % vs. 32 %), cigarette smoking (16.8 % vs. 14 %), and exercising (57.9 % vs. 55 %) [40].

We found that regular alcohol use coexisted with increased emotional exhaustion and depersonalization. This was likely similar in US physicians [19] and in Danish physicians for whom the greatest effect was observed in the DP dimension [41]. Our data could not support the findings of the study in Kazakhstan cardiologists [20], which showedthat using alcohol resulted in lower PA burnout than never alcohol users and were in contrast with the results of the studies in Hong Kong public doctors [18, 42] and Taiwan hospital workers [43], as these studies did not show any correlation between the use of alcohol and burnout. The interaction of burnout with smoking may be more considerable. We revealed that smoking was negatively associated with all burnout dimensions. Such an association of burnout and smoking may seem plausible and was found in other studies both in the general analysis and of selected burnout dimensions [21, 44, 45]. With regard to the interaction of burnout with smoking, several authors [21, 45] considered smoking as a predictor of feelings of stress and exhaustion related to work problems, while another study [20] showed that smoking did not predict job-associated burnout among doctors and nurses. Finally, our study showed a significant association of burnout with substance abuse and taking medications for sleep disorders among dentists in Lithuania. Several studies have also confirmed the substantial link of burnout with illegal substance use [19, 46] and taking medications for sleep disorders [21, 47]. However, our study showed a substantial interaction between these lifestyle factors. Individuals who reported regular use of both illicit substances and medications had significantly lower EE and DP sum scores. This is somewhat a surprising finding, but may prompt further examination to understand the underlying mechanism of such an association.

The results of the present study may suggest that dentists in Lithuania cope with job burnout by seeking support from family or friends, using different cognitive strategies (e.g., visiting a theatre, concerting, listening to music, reading nonmedical literature, engaging in art, etc.), and through leisure activities. The most common methods of leisure activities in the Lithuanian dentist population were playing sports and spending time in nature. Playing sports, for example, was positively associated with diminishing burnout in all three dimensions, as dentists who regularly played sports had significantly lower EE and DP sum scores and had better assessments of PA. Similar to the findings of our study, the protective effects of exercise on burnout have been seen in some studies in the USA on medical students [48], Germany on employees [49], Taiwan on hospital workers of different specialties [43], and Hong Kong on public doctors [18] and medical students [50], although there are studies to the contrary [42]. There is evidence that physical activity has a huge potential to enhance physical health and wellbeing [51], so it should be assumed that any kind of physical activity is a great way to prevent burnout [52]. It is likely that adequate sleep duration is also an important factor in reducing burnout [43, 47, 50]. In our study sleep in free time was associated with a lower DP sum score. In general, passive rest should be viewed negatively, but extra minutes of sleep are an important factor to reduce stress and fatigue of the doctor after a busy day [52]. Our study also identified that all dimensions of burnout were positively associated with eating a favorite food. However, the study could not reveal how healthy this food was. No previous studies have directly investigated the association between eating factors and burnout, yet Alexandrova-Karamanova et al. (2016) reported that burnout was significantly positively associated with higher fast food consumption [14]. We can still argue that exercise, sleep and diet are three pillars of lifestyle affecting dentists’ job burnout. Several recent studies have suggested that improving all three these pillars may be a better way to improve both physical and mental health [53].

## Limitations

Our study had several limitations. First, approximately one-third of the subjects in the study sample were interviewed online. These subjects did not form a randomized sample because dentists in the country do not have equal access to using the Internet or equal willingness to connect to a Facebook account.

Second, we analyzed burnout status based on the self-reported MBI questionnaire, so it may be biased. The literature [54] indicates that subjects with a high level of burnout might have felt the questions too sensitive and thus been unwilling to participate in the survey; in contrast, those who were more prone to answer the questionnaire were also more likely to experience burnout or overestimate burnout symptoms.

Third, some caution should be employed when generalizing the results to other professional groups or populations of dentists in other countries, as different labor employment systems may have different personal requirements and pressures, and the impact of lifestyle and relaxation factors on burnout may vary from culture to culture.

Fourth, the cross-sectional design of our study limits the validity of its findings, as the significant associations between burnout dimensions and other factors can be fully explained in longitudinal studies. However, with respect to the aim of our study, only a few longitudinal studies that have confirmed a positive effect of healthy relaxation (e.g., physical activity) in reducing burnout can be presented [48, 55].

Finally, in the survey, we collected a limited number of lifestyle and relaxation factors. Future studies with a larger number of lifestyle and relaxation factors would be beneficial to gain a more complete picture of burnout among Lithuanian dentists.

## Conclusions

The results confirmed a significant negative association of the burnout dimensions with unhealthy lifestyle factors (e.g., regular consumption of alcohol and smoking, drug abuse) and a positive association with some forms of active relaxation (e.g., playing sports), spending time with family or friends, using different cognitive strategies (e.g., visiting a theatre, concerting, listening to music, reading nonmedical literature, engaging in art). Therefore, burnout prevention among dentists in Lithuania should target specific lifestyle and relaxation improvement strategies including avoidance of the use of legal and illegal substances and promotion of sports and other active lifestyle measures.

## Data Availability

The dataset supporting the conclusions of this article is available from the corresponding author on reasonable request.

## Abbreviations

MBI: Maslach Burnout Inventory
EE: Emotional exhaustion
DP: Depersonalization
PA: Personal achievement
